# The mean–variance relationship reveals two possible strategies for dynamic brain connectivity analysis in fMRI

**DOI:** 10.3389/fnhum.2015.00398

**Published:** 2015-07-14

**Authors:** William H. Thompson, Peter Fransson

**Affiliations:** Department of Clinical Neuroscience, Karolinska InstituteStockholm, Sweden

**Keywords:** brain connectivity, resting-state, fMRI, dynamics, signal variance, mean

## Abstract

When studying brain connectivity using fMRI, signal intensity time-series are typically correlated with each other in time to compute estimates of the degree of interaction between different brain regions and/or networks. In the static connectivity case, the problem of defining which connections that should be considered significant in the analysis can be addressed in a rather straightforward manner by a statistical thresholding that is based on the magnitude of the correlation coefficients. More recently, interest has come to focus on the dynamical aspects of brain connectivity and the problem of deciding which brain connections that are to be considered relevant in the context of dynamical changes in connectivity provides further options. Since we, in the dynamical case, are interested in changes in connectivity over time, the variance of the correlation time-series becomes a relevant parameter. In this study, we discuss the relationship between the mean and variance of brain connectivity time-series and show that by studying the relation between them, two conceptually different strategies to analyze dynamic functional brain connectivity become available. Using resting-state fMRI data from a cohort of 46 subjects, we show that the mean of fMRI connectivity time-series scales negatively with its variance. This finding leads to the suggestion that magnitude- versus variance-based thresholding strategies will induce different results in studies of dynamic functional brain connectivity. Our assertion is exemplified by showing that the magnitude-based strategy is more sensitive to within-resting-state network (RSN) connectivity compared to between-RSN connectivity whereas the opposite holds true for a variance-based analysis strategy. The implications of our findings for dynamical functional brain connectivity studies are discussed.

## Introduction

In functional brain connectivity analysis we implicitly make the assumption that a temporal correlation between two regions (or alternatively, nodes) is indicative of an interaction between them. As it is often the case, measures of connectivity are derived by computing the correlation between signal intensity time-series in their entirety, that is, we achieve static measures of brain connectivity. In the case of static connectivity analysis, the underlying assumption is straightforward: a higher magnitude of correlation implies a larger degree of interaction between separate brain regions during the time span of measurement. This view of assessing functional brain connectivity has proven fruitful to identify functional networks during both resting-state and task conditions (Damoiseaux et al., 2006; Fox and Raichle, 2007). Moreover, resting-state networks (RSNs) are characterized by a strong degree of within-network connectivity, and in some cases, negative between-network connectivity (Fox et al., 2005; Fransson, 2005). Thus, at a macroscopic level, studies of functional connectivity together with previous work on the brain’s anatomical circuitry form the foundational stones for the notion that the flow of information in the brain is both segregated and integrated (Sporns, 2011; Bullmore and Sporns, 2012).

Recently, interest has been focused toward the dynamical aspects of brain connectivity (for a recent review, see Hutchison et al., 2013). It may seem appealing to apply the same kind of thinking as described above to the case of dynamical connectivity: if we find that the magnitude of correlation between two brain regions is large at a certain point in time, we assume that it would be a good candidate for a point in time when an interaction (i.e., a presence of an edge between the two nodes/regions, expressed in the commonly used graph–theoretical jargon) between the two regions occur. Thus, the procedure of identifying time-points of interactions (a presence of edges) between brain regions can be performed by searching for connections between brain regions that have large magnitude of correlation at each time-point. Obviously, this could be done by simply applying a global cut-off threshold which dictate that all correlation values above the chosen threshold (which could be defined statistically) is to be considered to constitute a significant edge and hence a time point when an interaction between nodes is assumed to occur. While thresholding of edges that is based on the absolute magnitude of correlation values seems to be a reasonable approach, an alternative approach to the problem of identifying dynamical changes in brain connectivity is available. Rather than relying on an approach that relies on the absolute magnitude of correlation coefficients *per se*, one can think of a shift of focus from magnitude-based thresholds toward examining fluctuations in correlation values over time. This view may at first seem less intuitive, but as will be shown in the examples given below, we argue that just as a large magnitude of correlation is indicative of brain connectivity, so might fluctuations in correlation over time considered to be of interest. The difference in connectivity between groups (e.g., between conditions or patient groups) looks at difference in connectivity across groups in much the same way that the fluctuation approach considers differences in functional connectivity across the time-series. We argue that the pursuit of locating the largest magnitudes of correlation as well as finding the largest fluctuations in correlation are both reasonable strategies and that they both meet the underlying assumptions regarding dynamic functional connectivity.

However, the two approaches to investigating dynamical brain connectivity may not necessarily lead to different results and thus be analogous strategies. For example, it is conceivable that fluctuations, or equivalently variance, in the time-series of correlation may be large while the magnitudes throughout the time-series remain relatively high. It is worthwhile to think of this situation in the context of Taylor’s Law (Taylor, 1961), first observed in ecological studies, which is a phenomena that postulates that the variance of data often scales positively with its mean. If the observation of Taylor (1961) is applicable to brain connectivity time-series, then the two analysis strategies would tend to produce similar information. To find out if the two approaches outlined here will lead to different results one needs to consider the relationship between the mean and the variance of a connectivity time-series. In this brief article we consider these two possible analysis methods by testing for the mean–variance relationship of connectivity time-series in fMRI using the sliding window method. We find that the two analysis strategies yield different information and that the amplitude-based approach is best suited to analyze within-network connectivity (i.e., the segregation of information in the brain) while the variance-based approach is best suited to analyze between-network connectivity (i.e., the integration of information in the brain).

## Materials and Methods

Resting-state fMRI data from 48 healthy subjects contained in the Beijing eyes open/eyes closed dataset available at http://fcon_1000.projects.nitrc.org/indi/IndiPro.html (Liu et al., 2013) were analyzed. Subject age ranged from 19 to 31 years (24 female). fMRI resting-state data were collected at 3 Tesla, TR = 2000 ms, TE = 30 ms. Each functional volume comprised 33 axial slices (thickness/gap = 3.5/0.7 mm, in-plane resolution = 64 × 64, FOV = 200 mm × 200 mm). Further details regarding the scanning procedure are given in Liu et al. (2013). Each imaging session consisted of three functional runs that each comprised 240 image volumes. Only fMRI data collected during the eyes open condition was used in this study. Resting-state fMRI data from two subjects were rejected due to incomplete data, leaving fMRI data acquired in 46 participants to be included in the final analysis. fMRI data was pre-processed using Matlab (Version 14b, Mathworks, Inc.), the CONN (Whitfield-Gabrieli and Nieto-Castanon, 2012) and SPM8 (Friston et al., 1995) Matlab toolboxes. Resting-state fMRI data was first realigned and subsequently normalized to the EPI MNI template as implemented in the SPM software platform. All functional images were spatially smoothed using a Gaussian filter kernel (FWHM = 8 mm). Image artifacts originating from head movement were handled using the image scrubbing procedure using the ART toolbox (www.nitrc.org/projects/artifact_detect/). Signal contributions from white brain matter, cerebrospinal fluid, and micro head-movement (six parameters) were regressed out from the data. After the regression, fMRI data was bandpassed (0.008–0.1 Hz), linear detrended, and despiked.

Resting-state fMRI signal intensity time-courses from 264 regions of interest (ROI) across the entire cortex and sub-cortical regions extracted and used in the subsequent analysis (further details regarding the parcellation scheme are given in Power et al., 2011). Each ROI was a defined as a sphere with a radius of 10 mm. To compare within- versus between-RSN connectivity, data from the 264 ROIs were divided into 10 RSNs (Auditory, Saliency, Sensorimotor, Fronto-parietal attention, Dorsal attention, Ventral attention, Subcortical, Cingulo-opercular, Visual, and Default mode Networks, see Cole et al., 2013 for further details). The spatial localization of the ROIs and their assignment to RSNs are shown in Supplementary Figure S1, along with the static functional connectivity matrix computed using the Spearman rank correlation coefficient. A sliding time-window consisting of 45 time-points (90 s) was used for the dynamical connectivity analysis which is comparable with the recent suggestions of “rules of thumb” regarding the window length for sliding window analysis (Leonardi and Van De Ville, 2015; Zalesky and Breakspear, 2015). For each time-window, the Spearman rank correlation coefficient between all ROI combinations was calculated. Subsequently, the sliding-window was slid 1 TR over the data, resulting in a ROI × ROI × time connectivity matrix time-series, for each subject, with the dimensions 264 × 264 × 240. For each subject and pair of edges, we calculated the mean and variance of the connectivity time-series. The mean and variance were then averaged over all subjects. This left us with 34716 unique edges for which we had computed both the mean and the variance over the connectivity time-series (196 time-points, reduced from 240 due to the sliding window size. This is because that the sliding window that would begin at the 197th time-point could not have a window length of 45). Finally, the mean and variances for all edges were then correlated with each other to test for Taylor’s Law using a Spearman test.

## Results

### An Illustrative Example of the Magnitude- and Variance-Based Approach to Study Dynamic Functional Connectivity

**FIGURE 1:**
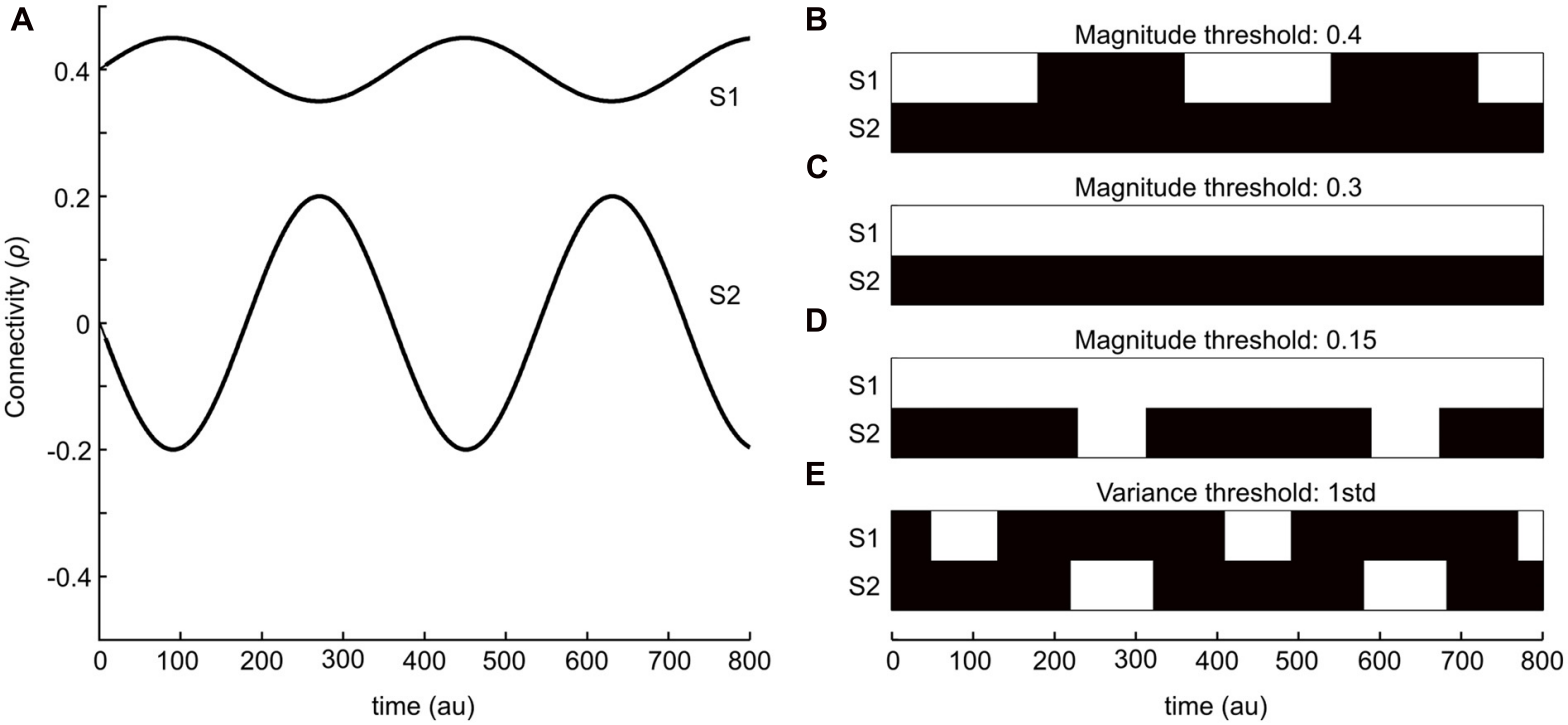
**A simple example that serves to illustrate the two kinds of dynamical functional connectivity analyses analysis described in this study. (A)** Two sinusoidal curves (S1 and S2, in anti-phase with respect to each other) that represents simulated fluctuations in connectivity (correlation) values between two brain regions in time. **(B–E)** Isolating time-points where an edge is considered “relevant” or “present” (shown as white patches) based on either magnitude or variance-based thresholding strategies. **(B–D)** Shows the results from using an absolute magnitude threshold at 0.3 **(B)**, 0.2 **(C)**, and 0.15 **(D)**. The panel in **(E)** shows a threshold strategy based on the variance of fluctuations within each time-series (1 SD). The variance-based threshold is able to isolate peaks in fluctuations for both time-series S1 and S2.

To illustrate the two strategies outlined in the introduction, we start by providing a simple schematic example. **Figure 1A** shows two sinusoidal curves that represent two ideal time-series for the degree of connectivity (correlation) over time. One of the time-series (S1) has, on general, a larger mean whereas the variance throughout the time-course is low. The other time-series (S2) has on the other hand a much lower mean value of correlation, but in this case the variance is substantially larger. Thus, the connectivity times-series plotted in **Figure 1A** is meant to be viewed as an illustrative example for putative outcomes of the temporal evolution of changes in brain connectivity between two different pairs of brain regions (nodes) during the length of a typical fMRI session. The binary graphs shown in **Figures 1B–D** depict the results by applying the magnitude-based strategy, including three different choices of cut-off thresholds (0.4, 0.3, and 0.2, respectively), to S1 and S2. Black patches denotes periods in time that by the magnitude-based analysis strategy were deemed to lack a significant degree of connectivity (i.e., an absence of an edge) between the two regions, whereas white patches denotes periods in time that has a presence of an edge. The binary graph shown in **Figure 1E** shows result from using the variance-based method (threshold set to 1 STD). Let us first consider the case of the magnitude-based approach. A cut-off threshold set to 0.4 applied to S1 and S2 as shown in **Figure 1B** results in a presence of connectivity (i.e., a presence of an edge) during the peaks of connectivity for S1 and an absence of connectivity during its troughs. No connectivity at all is detected for S2 at this choice of cut-off threshold. If we then consider the case of lowering the cut-off to 0.3 (**Figure 1C**), we get as a result that a significant degree of connectivity is present throughout the entire sampled time-window for S1 and, again, no connectivity at all is detected for S2. By setting the cut-off to an even lower value (0.15, **Figure 1D**), we arrive at the result that a significant degree of connectivity for S1 is considered to be present at all times, whereas we now also pick up a presence of connectivity during the peaks in S2.

Let us now turn our attention to the variance-based approach shown in **Figure 1E**, which by definition assesses the degree of connectivity in S1 and S2 independently from each other. The threshold was here set to 1 SD greater than its mean for each time-series. Hence, the variance-based approach to analyzing dynamic changes in connectivity takes into consideration that the degree of connectivity in S2 varies from -0.2 to 0.2 – a difference of 0.4, which according to our initial assumption represent a large change in interaction between two brain regions (albeit the interaction’s magnitude is still less than for S1, but the change in connectivity is larger than any change in S1). In other words, the variance-based approach finds time-points when the connectivity value is larger than usual which as a result detects time-points for which there is a peak in connectivity in S1 but not in S2 and vice versa. So, from this simple example it is evident that the two approaches to detect dynamical changes in connectivity might yield different results.

### The Temporal Mean Scales Negatively with the Variance for rs-fmri Correlation Time-Series

**FIGURE 2:**
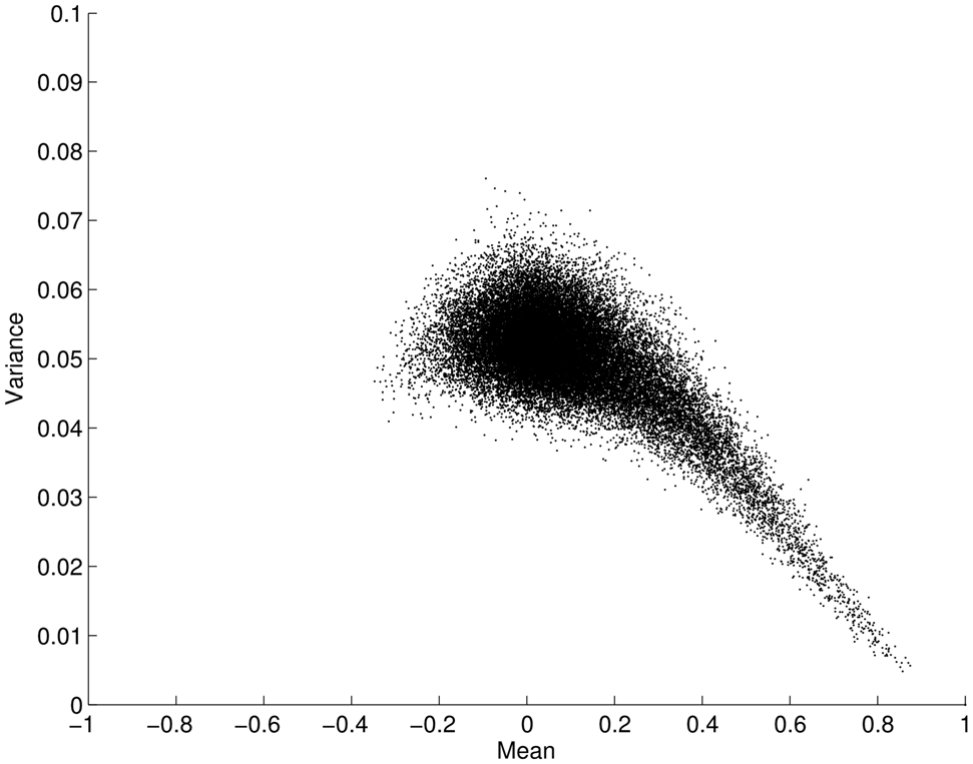
**Plot showing the mean versus the variance for fMRI connectivity time-series obtained during an eyes open condition (using a sliding window technique on a total of 34716 edges, averaged across subjects).** The mean of each edge regions of interest (pairwise ROI–ROI correlations over time) is plotted against its variance. Each point in the plot represents the mean and variance for a single ROI–ROI correlation (edge) across time. The plot shows that the mean (*x*-axis) is negatively correlated with its variance (*y*-axis; ρ = -0.5003; *p* < 0.001). Error bars show the SD.

We now leave our simulated example and turn our attention to the resting-state fMRI data. When considering the two approaches described above it is a pertinent question to ask whether the mean connectivity over time positively correlates with the variance as previously suggested by Taylor’s law. **Figure 2** shows the variance and mean in correlation over the time series for all connections (34716 edges) averaged over all subjects. Contrary to the situation posed by Taylor’s law, we found an opposite effect between the mean and variance for correlation time-series, i.e., the mean scaled negatively with variance (ρ = -0.5003; *p* < 0.001). This result clearly suggests that the two analysis approaches outlined in **Figure 1** will yield different results when applied to fMRI data. That is, a thresholding approach based on the connectivity variance parameter will lead to stronger emphasis on edges that, in general, have a lower mean. However, it is important to remember that we are analyzing connectivity time-series for which the maximum value is 1. This in turn leads to a restriction on the maximum variance that is allowed for a connectivity time-series that has a high mean correlation magnitude through the time-series. Given this, it is not overly surprising that the mean scales negatively with variance. However, there is no built-in mechanism that stipulates that the variance with a lower mean should have a larger degree of variance. Hence, this is a non-trivial result.

### Within-Network Connectivity Has a Higher a Mean and Lower Variance in Connectivity Values Compared to Between-Network Connectivity

**FIGURE 3:**
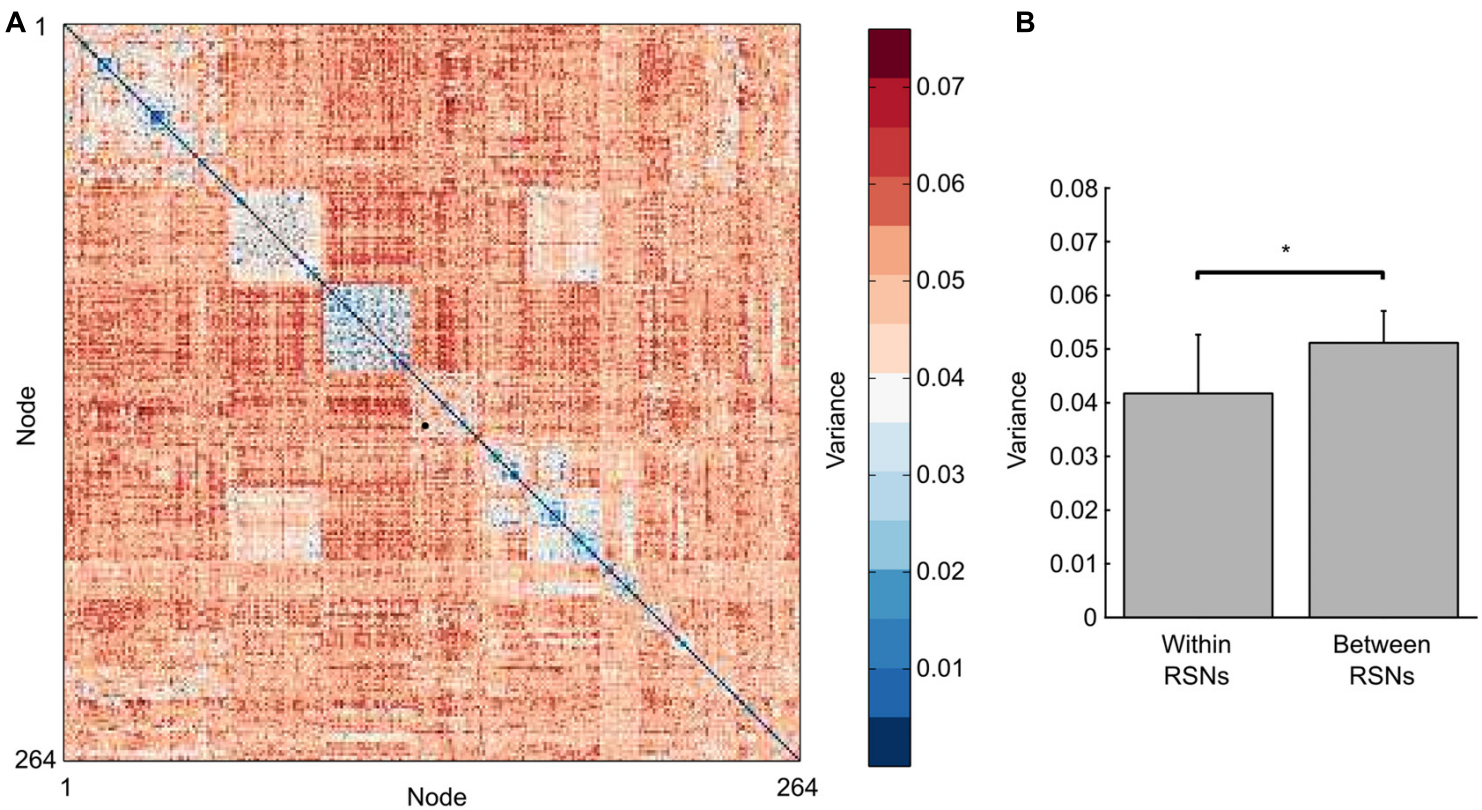
**Variance in the degree of edge (pairwise ROI–ROI correlations) connectivity over time is higher for between-RSN edges compared to within-RSN edge connectivity. (A)** shows in a connectivity matrix format the average variance of the connectivity time-series for each node-node pairing (averaged across subjects). Nodes are ordered according to the resting-state networks defined in Power et al. (2011) and Cole et al. (2013). The squares along the diagonal represent within-RSN edges, which tend to have a lower degree of variance than between-RSN edges located further away from the diagonal. **(B)** shows that the average variance for all within-RSN connectivity time-series has a significantly higher variance than between-RSN connectivity time-series (*t*-test, *p* < 0.001).

Given numerous previous studies that have employed static measures of brain connectivity to show a consistent set of RSNs in the human brain, one would expect that the degree of connectivity within-RSNs, per definition, have on average a high mean throughout the time-series. Our results shown in **Figure 2** suggest that in addition to having a high mean in correlation values, within-RSN connectivity should also display a lower variance. This indeed turns out to be the case, which is shown in **Figure 3A**, that plots the variance for all edges averaged across all subjects. The within-RSN connectivity, located along the diagonal of the edge-by-edge matrix, shows a reduced degree of variance compared to the variance of the between-RSN connectivity that is found further away from the diagonal. To demonstrate this observation further, we averaged all within-RSN edge variance and all between-RSN variance across all subjects and the results is shown in **Figure 3B**. We found a significant difference in variance, with a decrease in variance for within-RSN connections compared to between-RSN connections (*t*-test, two-tailed, *p* < 0.05). We conclude that between-RSN connectivity time-series display more variance compared to within-RSN time-series connectivity.

### Replication of Results Using Multiple Windows Lengths and the Pearson Correlation Coefficient

In order to show that the effects described here are not merely a byproduct of the specific window length chosen, we have replicated the results shown in Figures 2 and 3 in Supplementary Figure S2 using considerably shorter and longer window lengths (50 s and 130 s, respectively). Additionally, we show in Supplementary Figure S2 that the same results regarding the relationship between mean and variance also holds in the case of using the Pearson correlation coefficient rather than the Spearman rank coefficient to compute the functional connectivity matrix (sliding window length = 90 s).

## Discussion

In this study, we have described a conceptual difference in how dynamical changes in resting-state fMRI connectivity may be viewed and how to distinguish them analytically. We subsequently tested if the described conceptual difference is related to the phenomena known as Taylor’s Law. We found a negative scaling between the mean of the correlation time-series and its variance. Finally, we demonstrated that the within-RSN edges of connectivity have less variance in correlation values over time than between-RSN edges. From the results provided in this study, we conclude that there are, at least, two different strategies on how to perform dynamic functional connectivity analyzes, and that they are sensitive to very different properties of the data. The magnitude-based approach, in which a threshold is set at a certain global cut off point, will be relatively more sensitive to within-RSN connectivity. In contrast, the variance-based approach, for which the threshold is dependent on the variance of an edge’s time-series, will be relatively more sensitive to between-RSN fluctuations in correlation strength.

We consider this an important distinction that warrants consideration when planning and designing dynamic functional fMRI connectivity studies. For example, a popular strategy in dynamic functional connectivity is to cluster the connectivity matrices derived across the time-series using, for example, the *k*-means algorithm to derive “states” of brain connectivity. Importantly, the *k*-means algorithm will have a tendency to identify clusters along the dimensions with greater variance and, following the results shown here, will be less sensitive to fluctuations along those time-series with a higher overall mean – which in turn means less sensitivity to fluctuations within-RSN connectivity. While this does not necessarily have to be problematic, it is important to note that the *k*-means clustering method will have a tendency, given the same distributions, to partition the data along the between-RSN connectivity dimension when creating “states.” If this is not desirable outcome for the research question at hand, it would be possible to normalize or scale each connectivity time-series to mitigate the effect that clustering occur mainly along edges with higher variance (and thus lower mean connectivity). An alternative strategy would be to set a global threshold, statistically, or arbitrarily (e.g., keep the top 10% of all connections (edges) at each time-point), prior to using a clustering method or a distance metric and subsequently identify different states. This approach will be considerably more sensitive to within-RSN fluctuations since the higher variance of the other connections has been set to 0. So, the question is which strategy should be chosen? We believe that the answer to this question depends strongly on the research question at hand but an explicit distinction between these two methods has to be made.

It is worth reflecting over why between-RSN edges have a higher variance than the within-RSN counterparts along their connectivity time-series. We have identified two possible mechanisms, but we cannot rule out that others exist. First, the greater connectivity for between-RSNs edges, despite having an overall lower magnitude compared to the within-RSN edges, reflect moments in time of increased interaction between RSNs. Due to the nature of segregation between RSNs, the moments in time of increased interactions will be relatively rare. Thus they will be complimented by time-points when the degree of connectivity is lower, which in turn, will increase the overall variance. If so, the variance-based analysis strategy is best suited to identify moments or periods of time when information between networks is transferred. Second, it could be argued that the differences in between-RSN edge connectivity may simply reflect noise and that the higher variance for between-RSN connectivity may simply be due to a signal-to-noise property and thus suggest that the observed fluctuations in connectivity are driven by noise. But, if the latter argument is true, it would provide severe methodological difficulties to the usage of dynamic fMRI connectivity as a means to detect temporal trajectories of changes in neuronal connectivity, at least for the case of the variance-based method described here. While this is possibility which at the moment cannot be ruled out, it deserves to be mentioned that considerable work has been carried out to suggest that fluctuations in functional connectivity reflect changes in neuronal activity is valid assumption (Magri et al., 2012; Tagliazucchi et al., 2012; Chang et al., 2013; Thompson et al., 2013, 2014; see also Keilholz, 2014). Further work is needed to resolve this issue.

As a final remark, our connectivity time-series were derived by computing non-parametric Spearman rank correlation coefficients, which, especially considering rather short time-windows used, is reasonable and less sensitive to outliers. Moreover, we also replicated our results using the parametric Pearson correlation. However, it may be worth exploring if the observed negative relationship between the variance and mean of connectivity time-series occurs also for other metrics that are used to derive connectivity. It is possible to argue that the mean–variance relationship found here is merely a byproduct of the chosen method for dynamic functional connectivity, i.e., the sliding windows approach. Further investigations aimed to examine if the relationship holds with other proposed methods for dynamic functional connectivity is warranted. In sum, in this work we have conceptually disentangled two possible strategies for considering dynamic functional connectivity. Further, we have shown how the two approaches, while both valid with respect to the fundamental assumption of functional connectivity, yielded different results. We advise researchers of dynamic functional connectivity to consider these different approaches and decide which approach that fits their research question best.

## Conflict of Interest Statement

The authors declare that the research was conducted in the absence of any commercial or financial relationships that could be construed as a potential conflict of interest.
