# Supplementary material for: The mean–variance relationship reveals two possible strategies for dynamic brain connectivity analysis in fMRI
Source: Front Hum Neurosci. 2015 Jul 14;9:398. doi: 10.3389/fnhum.2015.00398 (PMC4500903; doi:10.3389/fnhum.2015.00398)
Supplement: Supplementary file 1 [file Image_1.PDF]

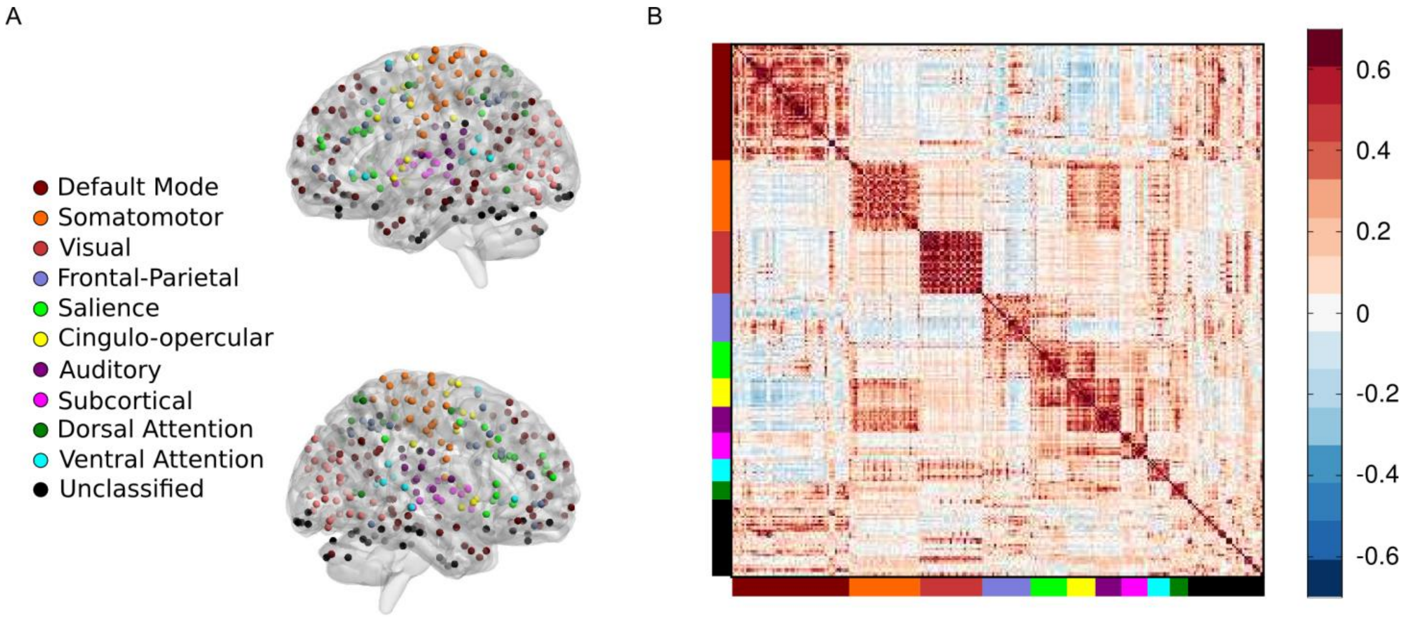

FIGURE S1 | (A) Spatial localization of region-of-interests (ROIs/nodes) used in the analysis and their corresponding resting-state network (RSN) membership. Nodes are defined using the template defined in Power et al. (2011). RSNs are defined using the template defined in Power et al. (2011), but reduced to 10 networks according to Cole et al. (2013). **(B)** Static functional connectivity matrix derived using the Spearman rank. Colors to the left and bottom of the matrix indicate assigned network of nodes as given in **(A)**.
