# Supplementary material for: The mean–variance relationship reveals two possible strategies for dynamic brain connectivity analysis in fMRI
Source: Front Hum Neurosci. 2015 Jul 14;9:398. doi: 10.3389/fnhum.2015.00398 (PMC4500903; doi:10.3389/fnhum.2015.00398)
Supplement: Supplementary file 2 [file Image_2.PDF]

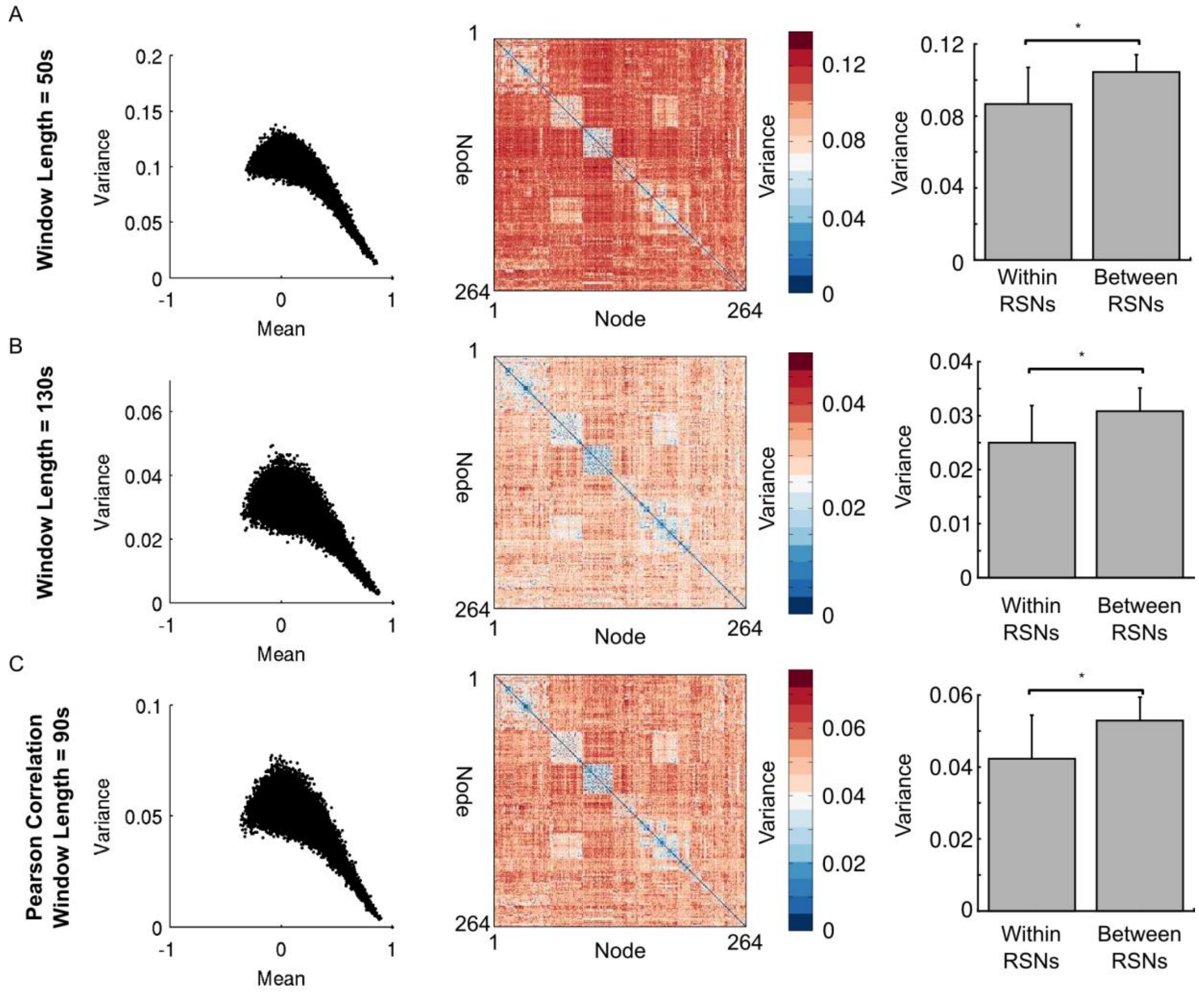

FIGURE S2 | Replication of the mean–variance relationship and the difference in variance between within- versus between-RSN connectivity for different choices of window length as well as the case of using the Pearson correlation instead of Spearman rank to compute correlation matrices. The results shown in the left column replicates the main results shown in Figure 2 but with a window length of 50 s (25 image volumes per window) **(A)** and 130 s (65 image volumes per window) **(B)**, respectively. The connectivity matrices results shown in the middle column replicates the findings shown in Figure 3A for the shorter **(A)** and longer window length **(B)**. The results shown in the right column replicates the difference in variance for within- versus between-RSN connectivity shown in Figure 3B (*t*-test, $p < 0.001$) for window durations of 50 s **(A)** and 130 s **(B)**, respectively. Error bars depict the SD. The results shown in **(C)** are based on using the Pearson correlation to derive functional connectivity rather than the Spearman rank that is used in the main text (window length = 90 s, same as in main text).
